# MASPC_Transform: A Plant Point Cloud Segmentation Network Based on Multi-Head Attention Separation and Position Code

**DOI:** 10.3390/s22239225

**Published:** 2022-11-27

**Authors:** Bin Li, Chenhua Guo

**Affiliations:** 1 School of Computer Science, Northeast Electric Power University, Jilin 132012, China; 2 Gongqing Institute of Science and Technology, No. 1 Gongqing Road, Gongqing 332020, China

**Keywords:** point cloud, plant phenotyping, point cloud segmentation, multi-head attention, attention separation, position code

## Abstract

Plant point cloud segmentation is an important step in 3D plant phenotype research. Because the stems, leaves, flowers, and other organs of plants are often intertwined and small in size, this makes plant point cloud segmentation more challenging than other segmentation tasks. In this paper, we propose MASPC_Transform, a novel plant point cloud segmentation network base on multi-head attention separation and position code. The proposed MASPC_Transform establishes connections for similar point clouds scattered in different areas of the point cloud space through multiple attention heads. In order to avoid the aggregation of multiple attention heads, we propose a multi-head attention separation loss based on spatial similarity, so that the attention positions of different attention heads can be dispersed as much as possible. In order to reduce the impact of point cloud disorder and irregularity on feature extraction, we propose a new point cloud position coding method, and use the position coding network based on this method in the local and global feature extraction modules of MASPC_Transform. We evaluate our MASPC_Transform on the ROSE_X dataset. Compared with the state-of-the-art approaches, the proposed MASPC_Transform achieved better segmentation results.

## 1. Introduction

Plant phenotype is to study how to measure the shape characteristics of plants, such as plant height, leaf organ size, root distribution, fruit weight, etc. These traits are closely related to the yield, quality, and stress resistance of plants. The study of plant phenotype has important value for agricultural modernization breeding [1], water and fertilizer managements of crop [2], and pest control [3].

In the process of plant phenotypic feature extraction, accurate segmentation of plant data according to different organs (stems, leaves, flowers, etc.,) is the premise of high-precision plant phenotype [4]. Plant organ segmentation technology based on 2D images has been very mature [5,6,7,8]. In recent years, with the development of LiDAR technology, more and more 3D spatial information of plants has been collected [9]. The plant point cloud contains the 3D spatial position, RGB color, normal vector, and other information of the collected object. Compared with 2D images, plant point cloud retains more spatial details and is not easily affected by occlusion, and can extract the plant structure more accurately.

By summarizing the existing segmentation methods of plant point clouds, we find that the existing methods have poor segmentation effect at the junction of different plant organs. For example, in the segmentation result in the fifth row and the first column in Figure 7, some stems are erroneously recognized as leaves. And this phenomenon is more obvious when the stem contacts the leaves. In the segmentation result in row 6 and column 2 of Figure 7, the part of the small calyx in the point cloud is erroneously divided into leaves. The reasons for the above segmentation errors are as follows: (1) for the plant segmentation task, the points belonging to the same plant organ are far from each other and are interwoven with the point clouds of other organs. For example, in Figures 5 and 6 the stems of the plants are almost distributed in the whole point cloud space and interweaved with other organs. The segmentation network often extracts the point cloud features of the whole plant without distinction, and does not mine the relationship of point clouds belonging to the same organ in the point cloud space. (2) Plant point clouds have the characteristics of disorder and irregularity, which will affect feature extraction.

To further improve the segmentation accuracy of plant point clouds, we use the Point Transformer [10] as the backbone of the proposed MASPC_Transform. The Point Transform uses the multi-head attentional mechanism in the process of local and global feature extraction. The multi-head attention mechanism can form associations between points of the same organ. And the multi-head attention mechanism is composed of multiple parallel self-attention mechanisms, which makes the whole feature generate multiple sub feature spaces and can extract feature information from multiple dimensions. However, the features of multi-head attention extraction may tend to be similar [11], that is, multiple attention heads establish connections for similar semantic point clouds at different positions in the point cloud space, but these point clouds may be located in the same area (for example, these point clouds may all be located on the same leaf of a plant). Therefore, we propose a multi-head attention separation loss based on spatial similarity, so that the attention positions of different attention heads can be separated from each other as much as possible, so as to establish a connection for point clouds that are distant in the point cloud space but belong to the same organ. In order to suppress the influence of point cloud disorder and irregularity on feature extraction, we added position coding network in the local and global feature extraction modules of MASPC_Transform.

The main contributions of this paper are summarized as follows:

1. We propose a plant point cloud segmentation network named MASPC_Transform, and evaluate its segmentation performance on the ROSE_X dataset.
2. We propose a loss function of multi-head attention separation based on spatial similarity. This loss can make the attention positions of different attention heads as dispersed as possible, and establish a connection for the point clouds that are far away but belong to the same organ, thus providing more semantic information for accurate segmentation.
3. In order to reduce the impact of point cloud disorder and irregularity on feature extraction, we propose a position coding method that can reflect the relative position of points, and use the position coding network in the local and global feature extraction modules of MASPC_Transform.

The rest of this paper is organized as follows. Section 2 introduces the related work of plant point cloud segmentation. Section 3 describes the detailed structure of MASPC_Transform. In Section 4, we evaluated the segmentation performance of MASPC_Transform on the ROSE_X dataset and analyzed the experimental results. The last part is the conclusion of this paper.

## 2. Related Work

Traditional methods achieve the segmentation of plant point clouds through geometric features [12]. These methods use geometric information such as point cloud edge points, smoothness, plane fitting residual [13], curvature gradient [14] to classify and aggregate each point. On this basis, the method of clustering and model fitting [15] is further applied to complete the segmentation of point cloud data. Lee et al. [16] developed an adaptive clustering method, which can segment the point cloud data of pine forest to manage individual pine trees. This method is suitable for different sizes of canopy, but it needs a lot of data for pre training. Tao et al. [17] completed the segmentation task of single tree by setting a reasonable spacing threshold by using the characteristics of different trees and combining the “growth” algorithm. Xu et al. [18] applied the traditional Dijkstra shortest path algorithm to the spatial point cloud to complete the separation of tree branches and leaves. Matheus et al. [19] fused a variety of algorithms to realize the recognition of geometric characteristics in tree point cloud, and combined with the shortest path algorithm to complete the segmentation of point cloud structure, which greatly improved the robustness of the algorithm. Li et al. [20] designed a new algorithm to more accurately estimate the inclination and azimuth of the blades in the point cloud, and constructed a new projection coefficient model. In the follow-up study, Li et al. [21] developed a new path discrimination method by improving Laplace’s shrinkage skeletonization algorithm to obtain the relevant parameters of the branch architecture. Traditional algorithms are easily affected by outliers and noise, which reduces the segmentation accuracy. The design of such algorithms often depends on the empirical design of geometric features, which are only effective for specific segmentation tasks.

Compared with traditional algorithms, deep learning methods are data-driven, do not need too many artificial design features, and have better performance. Currently, the deep learning methods that have been applied to point cloud segmentation include methods based on multi-view [22], voxel [23], and point cloud [24,25,26]. The method based on point cloud has the characteristics of directly processing point cloud and greatly retaining data information, so it has gradually become the mainstream research direction. Qi et al. [24] first proposed the network structure pointnet for directly processing point cloud data. This network proposed to use multilayer perceptron (MLP) with shared parameters to learn features and use symmetric functions to obtain global features. However, it has the problem that it cannot make full use of local information of points to extract fine-grained features. In order to solve this problem, an improved pointnet++ network [25] is proposed, which performs hierarchical and progressive learning on points from a large local area to obtain accurate geometric features near each point. In order to better extract the features of point clouds, Lee et al. [27] proposed an attention network, which can deal with disordered sets by adjusting the internal parameters of the network and can be used to extract the features of point clouds. Engel et al. [10] designed the Point Transformer network for point cloud segmentation, used multi-head attention in the network, and designed the SortNet structure to ensure the permutation invariance of extracted features.

Although great progress has been made in the research of deep learning segmentation algorithms for point cloud data, there are still few research on the segmentation of plant point cloud using deep learning methods. Wu et al. [28] adjusted the pointnet architecture to make the framework more suitable for processing the segmentation task of branches and leaves, and proposed a contribution score evaluation method. Jin et al. [29] made corn point cloud voxelized and applied convolutional neural network to voxelized data to complete a series of research work such as corn population segmentation and individual segmentation. Dutagaci et al. [30] provided valuable rosette data sets and provided benchmarks. Turgut et al. [31] verified the segmentation accuracy of various point based deep learning methods based on the work of Dutagaci [30], and studied the feasibility of three-dimensional synthesis model for training networks. Compared with other field point cloud segmentation tasks, plant point cloud segmentation is more challenging. This is because the stems, leaves, flowers, and other parts of plants are intertwined, resulting in the segmentation effect of existing segmentation methods is not ideal. The particularity of plant point cloud is that each part (organ) of the plant is very small and interwoven. This study proposes MASPC_Transform for segmentation of complex point clouds such as plant point clouds. In addition to the plant point cloud segmentation task, it is also applicable to the segmentation task of other point clouds with complex interwoven structures, such as forest point clouds [32].

## 3. Approach

### 3.1. Architecture of MASPC_Transform

**Figure 1:**
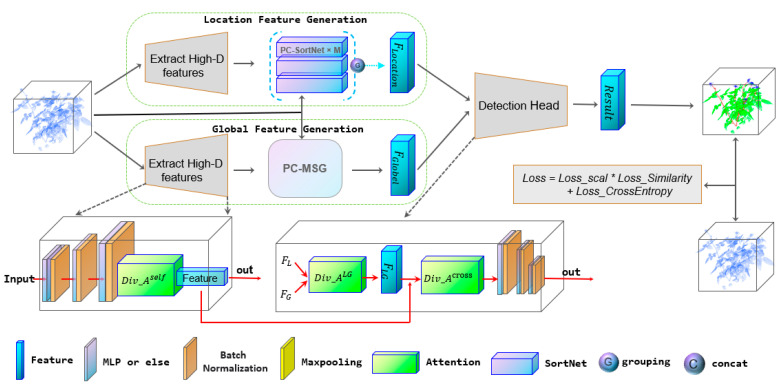
Architecture of MASPC_Transform.

The architecture of MASPC_Transform is shown in Figure 1. We use Point Transformer [10] as the network framework of MASPC_Transform. The difference between the proposed MASPC_Transform and the Point Transformer is that the proposed position coding network is used in the PC_MSG and PC_SortNet modules, and the proposed multi-head attention separation loss based on spatial similarity is added to the loss function of the entire network. MASPC_Transform includes feature extraction part and detection head. The feature extraction network has two branches: location feature generation and global feature generation. These two branches are responsible for extracting local and global features of plant point clouds. The global features (F_Globel_) and local features (F_Location_) are aggregated in the detection head and the segmentation results are obtained.

First, the plant point cloud is input to the location feature generation and global feature generation branches for processing. Both branches first extract the feature of point cloud. In the location feature generation, the PC-SortNet module can evaluate the importance of the features of different areas of the point cloud, and select the important features as the local features of the plant point cloud. In the global feature generation branch, the multi-scale grouped (MSG) feature extraction network can obtain the point cloud features of three scales to adapt to different sizes of plant organs. The features of the three scales are fused as the global features of the whole plant point cloud. We use the position coding proposed in this paper in PC_MSG and PC_SortNet modules. Position coding is discussed in detail in Section 3.1. In detection head, the global feature $F_{Globel}$ and the local feature $F_{Location}$ are associated and fused by the multi-head attention module. The multi-layer perceptron (MLP) in the detection head obtains the final segmentation result based on the fused features.

Multi-head attention [10] in MASPC_Transform is defined as follows:

(1) $$Multihead\left(Q,K,V\right)=\left(F_{sa}^{1}⨁\ldots ⨁F_{sa}^{i}\right)W^{O}$$

(2) $$A^{MH}\left(X,Y\right)=LayerNorm\left(S+\Phi \left(S\right)\right)$$

In Equation (1), *Q*, *K*, and *V* respectively represent the query matrix, key matrix, and value matrix of attention, and their matrix dimensions are $d_{k}$, $d_{k}$, and $d_{v}$. $F_{sa}^{i}=A\left(QW_{i}^{Q},KW_{i}^{K},VW_{i}^{V}\right)$ represents the features output by the *i*th attention head, $W_{i}^{Q},W_{i}^{K}\in {\mathbb{R}}^{d_{m}\times d_{k}}$, $W_{i}^{V}\in {\mathbb{R}}^{d_{m}\times d_{v}}$, and $W^{O}\in {\mathbb{R}}^{hd_{v}\times d_{m}}$ are the learnable parameters. The symbol ⨁ indicates that the features outputted by different attention heads are concatenated together. In Equation (2), LayerNorm is layer normalization [33]. S is defined as $S=LayerNorm\left(X+Multihead\left(X,\;Y,\;Y\right)\right)$, Φ is a network module with multiple MLPs, which is responsible for further feature extraction of S. $A^{MH}\left(X,Y\right)$ is the prototype of all multi-head attention in the network.

(3) $$A^{self}\left(P\right)=A^{MH}\left(P,P\right)$$

(4) $$A^{LG}\left(P,Q\right)=A^{cross}\left(P,Q\right)=A^{MH}\left(P,Q\right)$$

In Equations (3) and (4), *A^self^*, *A^LG^*, and *A^cross^* are derived from *A^MH^*. *A^self^* can perform the calculation of multi-head attention among all elements of *P*, while *A^LG^* and *A^cross^* can handle different sets *P* and *Q*, and perform the calculation of multi-head attention between the two sets.

We proposed a multi-head attention separation loss based on spatial similarity (loss in Figure 1). This loss acts on all the multi-head attention modules in MASPC_Transform. Therefore, we call the three attention modules that are affected by the proposed loss as $Div\_A^{self}$, $Div\_A^{LG}$, and $Div\_A^{cross}$. These three multi-head attention modules are responsible for establishing connections for similar features at different positions in the point cloud space. We will discuss the loss function of multi-head attention separation based on spatial similarity in Section 3.3.

### 3.2. Position Code

Plant point cloud data are a collection of a series of points in space. Point sets have the characteristics of disordered and irregular distribution, so we propose a unique point cloud position coding method. The position code contains the relative position information of each point and its adjacent points, so as to avoid the interference of the disorder of the point cloud on the feature extraction. Position code function *δ* is defined as follows:

(5) $$\delta =\theta \left(\overset{n}{\underset{i=1}{⋃}}\left(P_{i},(P_{i}-P_{i1}),\ldots \ldots ,(P_{i}-P_{ij})\right)\right)⨁\;\theta \left(P_{i},P_{i1},\ldots \ldots ,P_{ij}\right)$$

**Figure 2:**
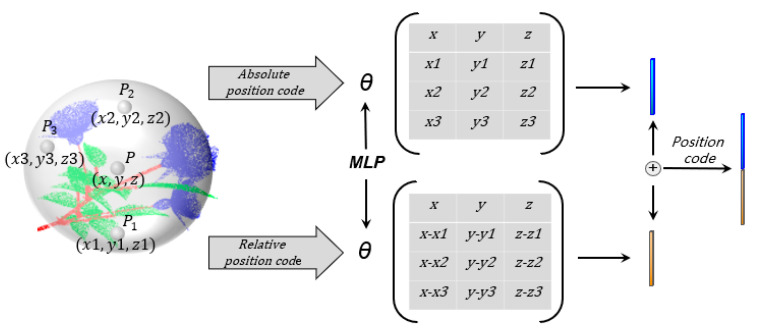
Position code network.

Suppose there are n points in the whole point cloud space. In Equation (5), $P_{i}$ is a point in a subspace after the ball query, $P_{i},P_{i1},P_{i2},P_{i3},\ldots ,P_{ij}\in P$, P is the set of all points in the subspace. $P_{i},(P_{i}-P_{i1}),\ldots \ldots ,(P_{i}-P_{ij})$ are the relative position codes of point $P_{i}$, ${⋃}_{i=1}^{n}()$ represents the relative position code of all points in the space. Function θ is a multi-layer perceptron (MLP) used for feature extraction of position code. The symbol ⨁ indicates that the obtained two features are concatenated. Equation (5) indicates that the position coding *δ* of the point cloud space is composed of the relative position code (RPC) and the absolute position code (APC) of each point in the space. The absolute position code of a point is the coordinates of the point cloud. The relative position code of a point is the difference between the coordinates of the point and all points in its subspace. The relative position code keeps a certain invariance to the disorder of the point cloud, and it reflects the relationship between a point and its adjacent points, which can make the feature contain more local information. The position code network is shown in Figure 2.

### 3.3. MSG and SortNet Based on Position Code Network

**Figure 3:**
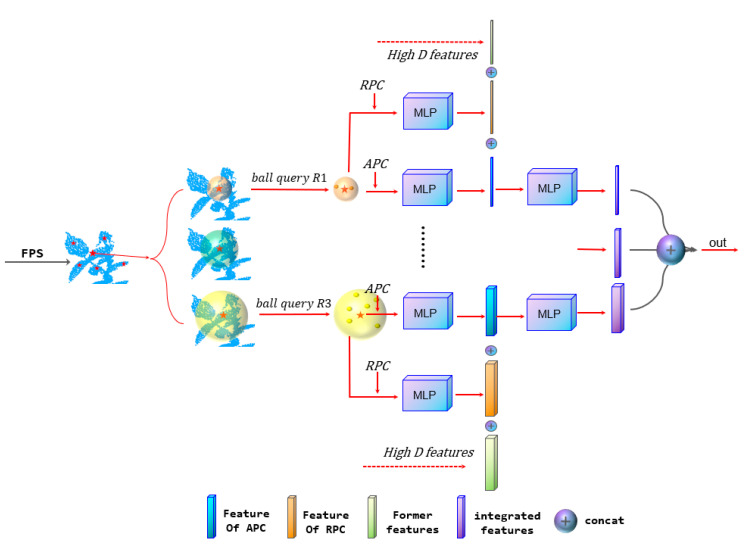
Position code in MSG.

In MASPC_Transform, we improved the MSG [10] in Point Transformer, and used the Position code-MSG(PC-MSG) module to extract global features. The structure of PC-MSG is shown in Figure 3. PC-MSG first takes the farthest point sampling (FPS), then the sampling point is taken as the center point, and three different radius are selected for ball query. According to the method in 3.1, the RPC of points is calculated in the subspace of each scale in PC-MSG. After that, the RPC features of each scale were extracted using MLP. In Figure 3, the orange rectangle represents the extracted RPC features of each scale, the blue rectangle represents the extracted APC features of each scale, and the high D features are the features extracted by the high-dimensional feature extraction network before the PC-MSG network. Finally, the RPC features, APC features, and high D features are concatenated together. Because the network structures of different scales in the MSG are the same, the feature extraction process of the second scale of the network is omitted in Figure 3.

**Figure 4:**
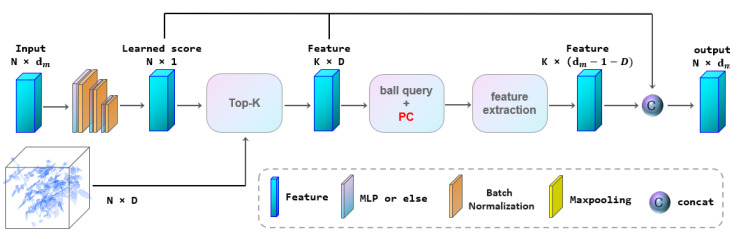
Position code in PC_SortNet.

We also improved SortNet in Point Transformer network [10], replacing SortNet with PC-SortNet with position code. As shown in Figure 4, in the PC-SortNet, the input features first pass through multiple MLPs, and its feature dimension is reduced to 1 dimension. This feature calculates a learnable importance score for each point in the point cloud space. After that, k points with the highest score are selected through the Top-k module. We take k points as the center of the ball query and extract the features of the region within the ball. We use a method similar to skip connect to concatenate the features of different stages. As shown by the Red PC in Figure 4, we use the position code proposed in Section 3.1 when querying the ball and extracting features.

### 3.4. Multi-Head Attention Separation Loss Based on Spatial Similarity

When multi-head attention is used for feature extraction, there is a possibility that the generated multiple attention spaces are similar [11], which will cause multiple attention spaces to overlap each other, resulting in repeated extraction in some areas and insufficient feature extraction in other areas. Therefore, we propose a multi-head attention separation loss based on spatial similarity, which makes each attention positions of the segmented network tend to be separated. Its definition is as follows:

(6) $$Separation\_Loss=-\frac{1}{n^{2}}\underset{F_{sa}^{i},F_{sa}^{j}\in F,i\neq j}{\sum }\frac{\left|\sum F_{sa}^{i}F_{sa}^{j}\right|}{{\left\|F_{sa}^{i}\right\|}_{2}{\left\|F_{sa}^{j}\right\|}_{2}}$$

In Equation (6), $F_{sa}^{i}$ and $F_{sa}^{j}$ are the different attention feature spaces of multi-head attention output, and F is the set of feature spaces output by the attention mechanism. The symbol $\left|\;.\;\right|$ represents the module of the matrix, ${\left\|\;.\;\right\|}_{2}$ denotes the 2-norm of the matrix. Equation (6) can calculate the average cosine distance of all output feature spaces. Cosine distance is an index to measure the difference of feature space in direction, so it can be used to evaluate the similarity of feature space. By dividing by $n^{2}$, we can make the calculated value tend to a reasonable range and avoid the difficulty of network training. Using a negative sign to indicate Separation_Loss penalizes network parameters that make $F_{sa}^{i}$ and $F_{sa}^{j}$ tend to be similar. We take the Separation_Loss as a part of the loss function and train the network, so that the attention features tend to be diverse. The loss function of MASPC_Transform is as follows:

(7) $$Loss\_CrossEntropy=-\underset{x}{\sum }\left(p\left(x\right)logq\left(x\right)+\left(1-p\left(x\right)\right)\log\left(1-q\left(x\right)\right)\right)$$

(8) $$Loss=Loss_{CrossEntropy}+Loss_{scal}\times Separation\_Loss$$

In Equation (7), $p\left(x\right)$ is the real classification probability distribution of the input point cloud, and $q\left(x\right)$ is the prediction probability distribution actually given by the network. Equation (7) depicts the difference between the classification result and the real value. The smaller the value of *Loss_CrossEntropy*, the more realistic the prediction given by the network. As shown in Equation (8), we used the *Loss_CrossEntropy* and *Separation_Loss* as MASPC_Transform’s loss function. Where *Loss_scal* is the weight of *Separation_Loss* in the loss function. Using the new loss function to train MASPC_Transform can make multiple attention feature spaces specific.

## 4. Experiment

### 4.1. Data Set

We evaluated the performance of MASPC_Transform on the ROSE_X dataset [30]. The ROSE_X dataset contains a total of 11 rose point cloud data. The rose point cloud data contain three semantic tags, namely, flower, leaf, and stem. The petals, calyx, and bud of rose are all marked as “flower” label, and the stem and petiole are all marked as “stem” label. We use nine rose point clouds to train the network, and the other two rose point clouds to test the segmentation performance of the network after training. We denoted the two roses used for the test as test_R1 and test_R2. Because the volume of a single rose point cloud is large and the number of points is large, and the amount of data that can be processed at a single time is limited, it is necessary to divide the point cloud into smaller blocks. We adopt the same blocking method as in [30], that is, the size and number of points of each block are as consistent as possible, and the structure within the block is as complete as possible. With this method, we divided the nine rose point clouds used for training into 596 point clouds and the two point clouds used for testing into 143 point clouds.

### 4.2. Implementation Details

For the model training, the Adam optimizer is used to update and optimize the network parameters. The initial learning rate is set to 0.001 and the batch size is 16. The GPU model is NVIDIA GeForce RTX 2080Ti, operating system is Ubuntu 18.04 LTS, CUDA version is 11.0. The proposed model is implemented in PyTorch with Python version 3.6. When training MASPC_Transform network, the input point cloud only contains three-dimensional X-Y-Z coordinates, and the number of input points is 2048.

### 4.3. Evaluation Methodology

We use the Intersection over Union (IoU) and Mean Intersection over Union (MIoU) to evaluate the performance of all networks. Where IoU is equal to the ratio of intersection and union between the predicted point set and the real point set, and MIoU represents the average value of IOU of all categories. The higher the values of these two indicators, the better the segmentation effect of the point cloud. The mathematical definition is as follows:

(9) $$IoU_{c}=\frac{TP_{c}}{TP_{c}+FP_{c}-FN_{c}}$$

(10) $$MIoU=\frac{\sum_{c}IoU_{c}}{k}$$

where $TP_{c}$, $FP_{c},$ and $FN_{c}$ are the number of positive samples of category C that have been correctly identified, the number of negative samples that have been misreported. and the number of positive samples that have been missed, $C\in \left\{Flower,\;stem,\;leaf\right\}$, k is the number of all categories.

### 4.4. Segmentation Results

In Table 1, we show the segmentation results of different segmentation networks on ROSE_X dataset, including PointNet [24], PointNet++ [25], DGCNN [34], PointCNN [35], ShellNet [36], RIConv [37], and the proposed MASPC_Transform.

In Table 1, we can see MASPC_Transform has the highest MIoU, and MASPC_Transform achieves the best segmentation results on both the flower and stem classes. As an improved version of PointNet, PointNet++ can flexibly extract local features by adjusting the neighborhood radius, and has the ability to extract the features of small organs of plants. So, it achieves the best segmentation results in leaf class. The IoU value of MASPC_Transform on the leaf class is slightly lower than that of PointNet++, but the MIoU value of MASPC_Transform is higher than that of PointNet++.

### 4.5. Visual Effects

**Figure 5:**
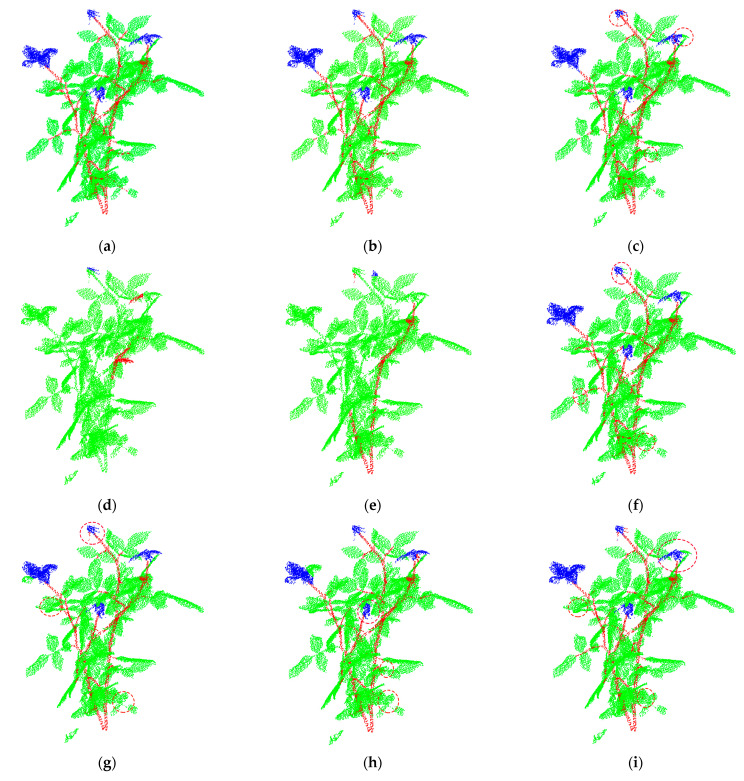
Segmentation result of each network on test_R1. (**a**) Ground Truth; (**b**) MASPC_Transform; (**c**) Point Transformer; (**d**) PointNet; (**e**) PointNet++; (**f**) DGCNN; (**g**) PointCNN; (**h**) ShellNet; (**i**) RICov.

**Figure 6:**
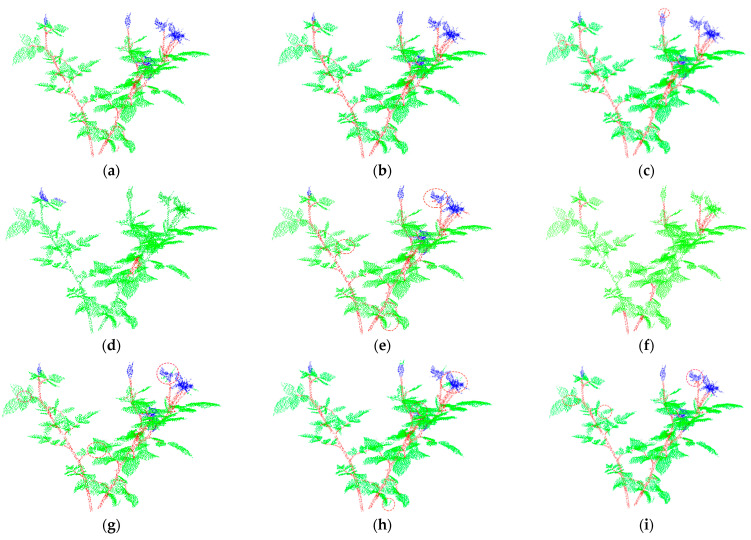
Segmentation result of each network on test_R2. (**a**) Ground Truth; (**b**) MASPC_Transform; (**c**) Point Transformer; (**d**) PointNet; (**e**) PointNet++; (**f**) DGCNN; (**g**) PointCNN; (**h**) ShellNet; (**i**) RICov.

Figure 5 and Figure 6 respectively show the segmentation results of different segmentation networks on test_R1 and test_R2. Figure 5a and Figure 6a are the ground truth of test_R1 and test_R2. In Figure 5a and Figure 6a, we can see that the stems, leaves, and flowers of the two plants are interlaced and occluded each other, which creates great difficulties for the segmentation algorithm. In Figure 5d,f and Figure 6d,f, we can see that PointNet and DGCNN hardly segment different plant organs. It can be seen from the area within the dotted circle in Figure 5 and Figure 6 that the segmentation ability of the comparison network (Point Transformer, PointNet++, DGCNN, PointCNN, ShellNet and RIConv) for details is inferior to that of MASPC_Transform. As shown in Figure 5c, Point Transformer mistakenly divides some petals into leaves. As shown in Figure 5e, PointNet++ mistakenly divided part of the calyx at the top into leaves and stems. As shown in Figure 5g, PointCNN mistakenly divided part of the calyx at the top into stems, and mistakenly divided the stems in the lowest red circle into leaves. As shown in Figure 5h, ShellNet mistakenly divided the calyx in the red circle into leaves. As shown in Figure 5i, RICov mistakenly divided some flowers in the top red circle into leaves. In Figure 6, there is also a case of false segmentation in the comparison network. The proposed MASPC_Transform has the best segmentation effect for the interlaced parts of different plant organs.

**Figure 7:**
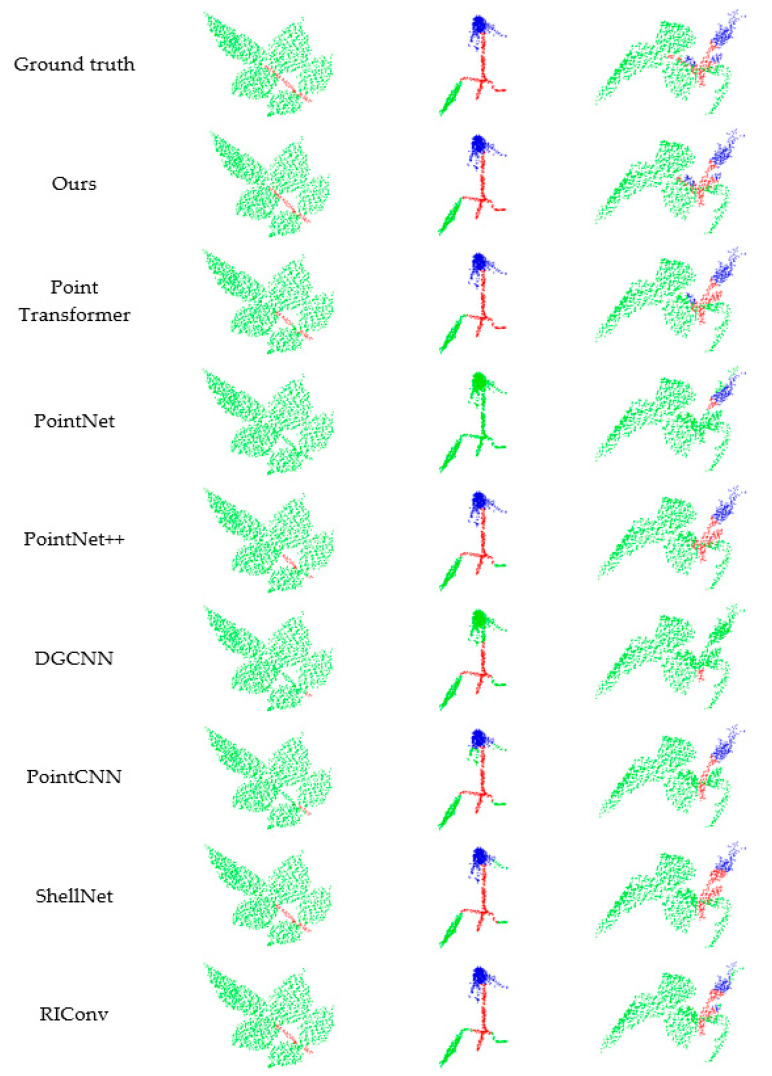
Segmentation rendering of different networks.

In order to show the segmentation effect of each method more clearly, we extracted some regions from the segmented plant point cloud and showed them more clearly in Figure 7. As can be seen from the first column in Figure 7, the objects to be segmented are leaves and stems. Among the segmentation results of all methods, the results corresponding to MASPC_Transform proposed by us are the most similar to ground truth. PointNet was failed to segment stems and leaves. DGCNN and PointCNN hardly segment the stem and leaf correctly. The stems segmented by PointNet++, ShellNet, and RIConv were shorter than those separated by MASPC_Transform, and they mistakenly divided the stems between two leaves into leaves. Point Transformer also mistakenly divides some stems into leaves at the intersection of leaves. In the segmentation results of the second and third columns of Figure 7, the MASPC_Transform also achieves the best segmentation effect.

It can be seen from the segmentation effect shown in Figure 5, Figure 6 and Figure 7 that the MASPC_Transform has the best segmentation effect. This is because the multi-head attention and the multi-head attention separation loss based on spatial similarity in MASPC_Transform establish a connection for the same kind of point clouds (point clouds with similar semantics) scattered in different regions of the point cloud space. In areas where multiple categories are interlaced, this association can help MASPC_Transform achieve better segmentation effect in detail.

### 4.6. Ablation Studies

Table 2 shows the results of our ablation studies on the ROSE_X dataset. In the ablation studies, we used the original Point Transformer [10] as the baseline. In Table 2, Without RPC represents a network that does not use RPC, but still uses our Equation (8) to train the network. Without Separation_Loss means that the proposed multi-head attention Separation_Loss is not used in the network, and only CrossEntropy is used to train the network. Note that RPC is used in the Without Separation_Loss network. The last column presents the experimental results of MASPC_Transform proposed by us. From the results shown in Table 2, we can see that the values of IoU and MIoU of MASPC_Transform are the highest. The IoU and MIoU of each category of MASPC_Transform without multi-head attention separation loss function and MASPC_Transform without relative position code are lower than those of MASPC_Transform, but better than the Point Transformer.

According to the experimental results in Section 4.4, the proposed MASPC_Transform outperforms the state-of-the-art approaches. The visualization results shown in Figure 6 and Figure 7 confirm the experimental results in Section 4.4. The visualization results of these comparison approaches for rose point clouds with interlaced stems, leaves, and flowers is not as good as MASPC_Transform. This shows that our multi-head attention separation loss can distract the attention positions of different attention heads as much as possible, and establish connections for point clouds that are far away but belong to the same organ. However, these comparison approaches do not have this ability, so that these segmentation networks believe that two flowers (stems or leaves) far away belong to different categories. The results of ablation studies verify the effectiveness of the multi-head attention separation loss (Separation_Loss) and position code (PC).

## 5. Conclusions

We propose a plant point cloud segmentation network named MASPC_Transform. In order to make the attention positions of different attention heads of MASPC_Transform as dispersed as possible, we propose a multi-head attention separation loss based on spatial similarity. In order to reduce the impact of point cloud disorder and irregularity on feature extraction, we use position coding in the local and global feature extraction modules of MARP_Transform. We evaluated the proposed MASPC_Transform on the ROSE_X dataset. The results of segmentation experiments show that MASPC_Transform network performs better than the state-of-the-art approaches. The results of ablation experiments demonstrate the effectiveness of the proposed position code and attention separation loss. Due to ROSE_X dataset is the only open source benchmark plant point cloud segmentation dataset, so the MASPC_Transform has only been tested on this dataset. If a new open source plant point cloud segmentation dataset appears, the MASPC_Transform should accept more tests.

## Figures and Tables

**Table 1:** Comparison of network segmentation effect indicators (%).

Evaluation	Category	Pointnet	Pointnet++	DGCNN	PointCNN	ShellNet	RIConv	Point Transformer	Ours
IoU	Flower	15.83	74.12	8.34	53.56	49.36	54.12	80.93	**83.32**
	Leaf	82.56	**95.36**	84.17	91.76	89.69	88.96	91.76	94.36
	Stem	5.27	77.69	24.97	70.89	54.78	35.79	74.99	**78.96**
MIoU	MIou	34.55	82.39	39.16	72.14	64.61	60.79	82.56	**85.52**

**Table 2:** Ablation study on ROSE_X dataset.

Evaluation	Category	Point Transformer	Without RPC	Without Separation_Loss	Ours
IoU	Flower	80.93	83.10	82.28	**83.32**
	Leaf	91.76	93.03	92.89	**94.36**
	Stem	74.99	77.64	76.71	**78.96**
MIoU	MIou	82.56	84.29	83.96	**85.52**

## Data Availability

Not applicable.

## References

[1] Crossa J., Fritsche-Neto R., Montesinos-Lopez O.A., Costa-Neto G., Dreisigacker S., Montesinos-Lopez A., Bentley A.R. (2021). The modern plant breeding triangle: Optimizing the use of genomics, phenomics, and enviromics data. Front. Plant Sci..

[2] Shi Y., Zhu Y., Wang X., Sun X., Ding Y., Cao W., Hu Z. (2020). Progress and development on biological information of crop phenotype research applied to real-time variable-rate fertilization. Plant Methods.

[3] Arbona V., Iglesias D.J., Talón M., Gómez-Cadenas A. (2009). Plant phenotype demarcation using nontargeted LC-MS and GC-MS metabolite profiling. J. Agric. Food Chem..

[4] Sun S., Li C., Chee P.W., Paterson A.H., Jiang Y., Xu R., Robertson J.S., Adhikari J., Shehzad T. (2020). Three-dimensional photogrammetric mapping of cotton bolls in situ based on point cloud segmentation and clustering. ISPRS J. Photogramm. Remote Sens..

[5] Sun S., Liang N., Zuo Z., Parsons D., Morel J., Shi J., Wang Z., Luo L., Zhao L., Fang H. (2021). Estimation of botanical composition in mixed clover–grass fields using machine learning-based image analysis. Front. Plant Sci..

[6] Aginako N., Lozano J., Quartulli M., Sierra B., Olaizola I.G. Identification of plant species on large botanical image datasets. Proceedings of the 1st International Workshop on Environnmental Multimedia Retrieval co-located with ACM International Conference on Multimedia Retrieval, EMR@ ICMR 2014.

[7] Grand-Brochier M., Vacavant A., Cerutti G., Kurtz C., Weber J., Tougne L. (2015). Tree leaves extraction in natural images: Comparative study of preprocessing tools and segmentation methods. IEEE Trans. Image Process..

[8] Yogeswararao G., Malmathanraj R., Palanisamy P. (2022). Fractional weighted nuclear norm based two dimensional linear discriminant features for cucumber leaf disease recognition. Multimed. Tools Appl..

[9] Li Y., Gao J., Wang X., Chen Y., He Y. (2022). Depth camera based remote three-dimensional reconstruction using incremental point cloud compression. Comput. Electr. Eng..

[10] Engel N., Belagiannis V., Dietmayer K. (2021). Point transformer. IEEE Access.

[11] Li J., Tu Z., Yang B., Lyu M.R., Zhang T. (2018). Multi-head attention with disagreement regularization. arXiv.

[12] Perez-Perez Y., Golparvar-Fard M., El-Rayes K. (2021). Segmentation of point clouds via joint semantic and geometric features for 3D modeling of the built environment. Autom. Constr..

[13] Li L., Sung M., Dubrovina A., Yi L., Guibas L.J. Supervised fitting of geometric primitives to 3d point clouds. Proceedings of the IEEE/CVF Conference on Computer Vision and Pattern Recognition.

[14] He Y., Kang S.H., Liu H. (2020). Curvature regularized surface reconstruction from point clouds. SIAM J. Imaging Sci..

[15] Xia S., Chen D., Wang R., Li J., Zhang X. (2020). Geometric primitives in LiDAR point clouds: A review. IEEE J. Sel. Top. Appl. Earth Obs. Remote Sens..

[16] Lee H., Slatton K.C., Roth B.E., Cropper W.P. (2010). Adaptive clustering of airborne LiDAR data to segment individual tree crowns in managed pine forests. Int. J. Remote Sens..

[17] Tao S., Wu F., Guo Q., Wang Y., Li W., Xue B., Hu X., Li P., Tian D., Li C. (2015). Segmenting tree crowns from terrestrial and mobile LiDAR data by exploring ecological theories. ISPRS J. Photogramm. Remote Sens..

[18] Xu H., Gossett N., Chen B. (2007). Knowledge and heuristic-based modeling of laser-scanned trees. ACM Trans. Graph..

[19] Vicari M.B., Disney M., Wilkes P., Burt A., Calders K., Woodgate W. (2019). Leaf and wood classification framework for terrestrial LiDAR point clouds. Methods Ecol. Evol..

[20] Li Y., Su Y., Hu T., Xu G., Guo Q. (2018). Retrieving 2-D leaf angle distributions for deciduous trees from terrestrial laser scanner data. IEEE Trans. Geosci. Remote Sens..

[21] Li Y., Su Y., Zhao X., Yang M., Hu T., Zhang J., Liu J., Liu M., Guo Q. (2020). Retrieval of tree branch architecture attributes from terrestrial laser scan data using a Laplacian algorithm. Agric. For. Meteorol..

[22] Su H., Maji S., Kalogerakis E., Learned-Miller E. Multi-view convolutional neural networks for 3d shape recognition. Proceedings of the IEEE International Conference on Computer Vision.

[23] Maturana D., Scherer S. Voxnet: A 3d convolutional neural network for real-time object recognition. Proceedings of the 2015 IEEE/RSJ International Conference on Intelligent Robots and Systems (IROS).

[24] Qi C.R., Su H., Mo K., Guibas L.J. Pointnet: Deep learning on point sets for 3d classification and segmentation. Proceedings of the IEEE Conference on Computer Vision and Pattern Recognition.

[25] Qi C.R., Yi L., Su H., Guibas L.J. (2017). Pointnet++: Deep hierarchical feature learning on point sets in a metric space. Adv. Neural Inf. Process. Syst..

[26] Li B., Zhu S., Lu Y. (2022). A single stage and single view 3D point cloud reconstruction network based on DetNet. Sensors.

[27] Kim B.N., Lee J.S., Shin M.S., Cho S.C., Lee D.S. (2002). Regional cerebral perfusion abnormalities in attention deficit/hyperactivity disorder. Eur. Arch. Psychiatry Clin. Neurosci..

[28] Wu B., Zheng G., Chen Y. (2020). An improved convolution neural network-based model for classifying foliage and woody components from terrestrial laser scanning data. Remote Sens..

[29] Jin S., Su Y., Gao S., Wu F., Hu T., Liu J., Li W., Wang D., Chen S., Jiang Y. (2018). Deep learning: Individual maize segmentation from terrestrial lidar data using faster R-CNN and regional growth algorithms. Front. Plant Sci..

[30] Dutagaci H., Rasti P., Galopin G., Rousseau D. (2020). ROSE-X: An annotated data set for evaluation of 3D plant organ segmentation methods. Plant Methods.

[31] Turgut K., Dutagaci H., Galopin G., Rousseau D. (2022). Segmentation of structural parts of rosebush plants with 3d point-based deep learning methods. Plant Methods.

[32] Krisanski S., Taskhiri M.S., Aracil S.G., Herries D., Turner P. (2021). Sensor agnostic semantic segmentation of structurally diverse and complex forest point clouds using deep learning. Remote Sens..

[33] Ba J.L., Kiros J.R., Hinton G.E. (2016). Layer normalization. arXiv.

[34] Zhang K., Hao M., Wang J., de Silva C.W., Fu C. (2019). Linked dynamic graph cnn: Learning on point cloud via linking hierarchical features. arXiv.

[35] Li Y., Bu R., Sun M., Wu W., Di X., Chen B. (2018). Pointcnn: Convolution on x-transformed points. Adv. Neural Inf. Process. Syst..

[36] Zhang Z., Hua B.S., Yeung S.K. Shellnet: Efficient point cloud convolutional neural networks using concentric shells statistics. Proceedings of the IEEE/CVF International Conference on Computer Vision.

[37] Zhang Z., Hua B.S., Rosen D.W., Yeung S.K. Rotation invariant convolutions for 3d point clouds deep learning. Proceedings of the 2019 International Conference on 3d Vision (3DV).

